# Cell Matrix Remodeling Ability Shown by Image Spatial Correlation

**DOI:** 10.1155/2013/532030

**Published:** 2013-07-14

**Authors:** Chi-Li Chiu, Michelle A. Digman, Enrico Gratton

**Affiliations:** ^1^Department of Developmental and Cell Biology, University of California, Irvine, CA 92697, USA; ^2^Laboratory for Fluorescence Dynamics, Department of Biomedical Engineering, University of California, Irvine, CA 92697, USA

## Abstract

Extracellular matrix (ECM) remodeling is a critical step of many biological and pathological processes. However, most of the studies to date lack a quantitative method to measure ECM remodeling at a scale comparable to cell size. Here, we applied image spatial correlation to collagen second harmonic generation (SHG) images to quantitatively evaluate the degree of collagen remodeling by cells. We propose a simple statistical method based on spatial correlation functions to determine the size of high collagen density area around cells. We applied our method to measure collagen remodeling by two breast cancer cell lines (MDA-MB-231 and MCF-7), which display different degrees of invasiveness, and a fibroblast cell line (NIH/3T3). We found distinct collagen compaction levels of these three cell lines by applying the spatial correlation method, indicating different collagen remodeling ability. Furthermore, we quantitatively measured the effect of Latrunculin B and Marimastat on MDA-MB-231 cell line collagen remodeling ability and showed that significant collagen compaction level decreases with these treatments.

## 1. Introduction

Extracellular matrix (ECM) remodeling through cell-ECM interactions is a critical step of many biological and pathological processes such as embryonic development [1], angiogenesis [2], wound healing [3], and cancer cell metastasis [4]. For instance, when cancer cells move through a dense ECM, they can generate actomyosin forces that deform the collagen fibers to push the cell through the ECM [5]. Reciprocally, the physical properties of the ECM including matrix structure, mechanics, and dimensionality can profoundly influence cellular behavior. In the case of cancer ECM which is usually stiffer than normal tissues, the compromised tensional homeostasis affects cell phenotype, Rho-dependent cell contractility and oncogene-mediated transformation [6]. Clinically, the increased matrix stiffness and ECM remodeling were observed in premalignant tissue, and this increase was shown to contribute to malignant transformation [7].

 Collagen, the most abundant protein in a mammalian body, is the major contributor to tissue mechanical properties. These mechanical properties have roots in collagen's microstructure, network organization, and orientation. Collagen has been used in a number of studies that aim to quantify cell-mediated ECM remodeling process and its mechanics. The measurement scale of ECM remodeling ranges from whole gel contraction assay at centimeter scale [8–11] to collagen properties at micrometer scale such as fiber diameter, fiber length, and pore size [12]. Also the organization of the collagen matrix in the proximity of the cells has been a subject of several investigations [13, 14]. Although these methods provide ways to describe the collagen matrix property changes around the cells and at the microscopic level, they lack the information of heterogeneity due to ECM remodeling at the mesoscale that is comparable to cell size. Alternatively, Stevenson et al. [15] attempted to quantify the collagen matrix compaction level in the pericellular region by creating an intensity contour map of a confocal collagen reflection image. This method, however, does not allow an absolute quantitative comparison of different images due to the dependence of intensity range of each image. 

Nonlinear microscopy techniques such as second harmonic generation (SHG) provide a noninvasive and label-free tool to image collagen fibers. Collagen has highly crystalline triple-helix structure that is not centrosymmetric, which makes it extremely bright in SHG. SHG does not involve the excitation of molecules; as a result, the molecules do not suffer the effects of phototoxicity or photobleaching. Since the first publication of SHG on collagen three decades ago [16], this technique has become a robust tool for imaging tissue structure in both *ex vivo* and *in vivo* preparations [4, 17–19]. 

In this study, we aimed to develop a quantitative method based on image spatial correlation of collagen SHG images to analyze collagen organization at mesoscale, that is, at the scale of 10–100 *μ*m, comparable to cellular size. Image spatial correlation provides an unbiased way for estimating the size of spatial organization. By applying image spatial correlation to collagen SHG images with proper resolution, it is possible to extract the collagen mesoscale organization information. During cell-mediated ECM remodeling, cells may generate collagen-dense areas through cell-ECM interactions that modify the density of collagen in the regions around the cells. We applied this method to estimate the mesoscale changes of SHG high intensity area size due to the presence of cells in the collagen matrix few days after seeding the cells. This parameter is referred to as collagen compaction level in this report, the same as Stevenson et al.'s definition [15]. Relevant to our methodology, applying image correlation spectroscopy to SHG image has been described by Raub et al. [20], which focused on quantifying collagen matrix pore size and fiber dimension from collagen. In contrast to the emphasis of the microscopic scale in the previous report, here we applied the spatial correlation technique to analyze the mesoscale structure of collagen and showed that the SHG image of collagen combined with image correlation spectroscopy is an effective means to assess the mesoscale properties of an *in vitro* ECM that involves cellular remodeling. We also emphasize that the image correlation method used in this work provides unbiased quantitative evaluation of the mesoscale collagen organization. This is important for comparison of different cell types and for the assessment of the effect of drugs treatment in regard to the large scale organization of the ECM. 

In this report, we compared the collagen matrix remodeling ability of two human breast cancer cell lines with distinct degrees of invasiveness, MDA-MB-231 and MCF-7, and fibroblast cell line NIH/3T3. Tumor cell invasiveness has been associated with increased contractile force generation [21], and ECM compaction level may reflect cell contractility. Our results suggested that cancer cells with high invasiveness have significantly more ability to remodel the collagen matrix, consistent with the previous report [21], while NIH/3T3 cell line moderately changed the collagen compaction level. Furthermore, the matrix remodeling ability of invasive cancer cell line can be altered through metalloproteinases (MMP) inhibitor or sequestering F-actin, suggesting that MMP and actin cytoskeleton affect collagen remodeling at the mesoscale.

## 2. Methods

### 2.1. Cell Culture in 3D Collagen Matrix

MDA-MB-231, MCF-7, and NIH/3T3 cell lines (American Type Culture Collection HTB-26, HTB-22, and CL-1658, resp.) were obtained from Dr. Wen-Hua Lee's lab in UC Irvine. Cells were cultured in Dulbecco's modified Eagle's medium (DMEM) with high glucose (Sigma, St. Louis, MO) supplemented with 10% (v/v) fetal bovine serum (FBS) at 37°C in a 5% CO_2_ humid incubator. 

Type I collagen with original concentration of 3.75 mg/mL was purchased from BD Biosciences (Franklin Lakes, NJ). Collagen was diluted with 10X PBS and water to achieve final concentration of 1X PBS and 2 mg/mL collagen. NaOH was added to neutralize collagen solution before mixing with cells. Cells in serum-free DMEM were mixed with collagen solution. The final concentration of 5 ∗ 10^4^ cells/mL cell-collagen mixture was added to 8-well Lab-Tek chambered coverglass with surface area 0.7 cm^2^ per well (Thermo Scientific, Rochester, NY).

Collagen was polymerized at 20°C for 1 hr and then at 37°C for 20 minutes. Full medium was applied after polymerization. Medium was changed everyday after collagen polymerization for both collagen-only and cell-collagen matrix to ensure minimum variation caused by evaporation.

Matrices were kept at 37°C, 5% CO_2_ incubator. Images were taken under room temperature after 1, 2, and 4 days of collagen polymerization. 

### 2.2. Pharmacological Treatment

 MDA-MB-231 cells were cultured in type I collagen matrix and applied pharmacological treatment everyday when changing the medium. The treatment was applied to the cell-collagen matrix for 4 consecutive days before imaging. The F-actin sequestering drug Latrunculin B (Molecular Probes, Eugene, OR) was used at 1 *μ*M final concentration. The matrix metalloproteinase inhibitor Marimastat (Tocris Bioscience, Bristol, United Kingdom) was used at 10 *μ*M final concentration.

### 2.3. 2-Photon Microscopy

To assess the degree of collagen matrix remodeling, collagen second harmonic generation (SHG) images were collected using LSM 710 (Carl Zeiss, Maple Grove, MN) with a 40 × 0.75 N.A. water immersion lens. The Mai-Tai laser (Newport, Irvine, CA) with emission at 900 nm was used for second harmonic generation. The collagen SHG propagating in backward direction was collected using a bandwidth filter (442–463 nm). Laser power and detector gain were fixed for all images taken. 

We conducted 4 independent experiments for the samples containing collagen only, collagen with MCF cell line, and collagen with NIH cell line. We did 8 independent experiments for the collagen with MB231 cell line, and 3 independent experiments for each drug treatment. For each independent experiment, we used 3 different regions for analysis. Each measurement has 10 *z*-stacks.

**Figure 1:**
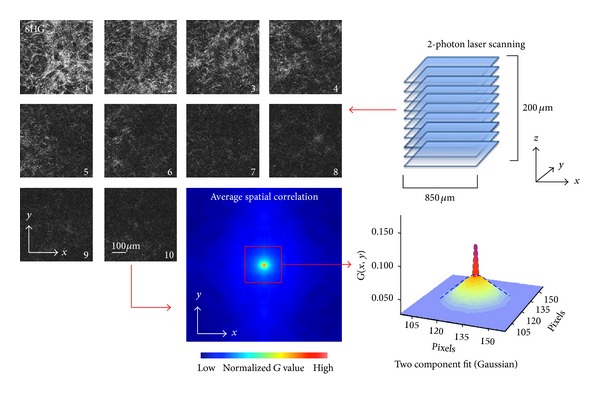
*Experimental workflow*—*Collagen remodeling analysis using image spatial correlation*. Upper right panel: collagen SHG images were acquired by 2-photon microscopy. 10 *z*-stack slices across 200 *μ*m depth from culture dish surface were collected. Each slice covers 850 *μ*m × 850 *μ*m. Upper left panel: an example of collagen SHG images from close to the culture dish surface (slice 1) to 200 *μ*m above (slice 10). The SHG intensity decreases with the increasing depth of acquisition. However, since image spatial correlation is not sensitive to average intensity change, image spatial correlation (1) was applied to each slice, and the results were averaged to produce one correlation image. Lower left panel: the spatial correlation image calculated from the SHG *z*-stack. The relatively symmetrical autocorrelation pattern indicates that with our sample preparation, collagen fibers were not preferentially aligned toward specific direction at the scale of the whole image. Lower right panel: the *x* and *y* cross sections from the spatial correlation image were fitted with two Gaussian components. One component has the standard deviation close to pixel size (green curve); the other component has broader standard deviation (blue curve), and its width varies based on the collagen compaction level.

For each measurement, as shown in Figure 1 upper right panel, *z*-stack SHG images with total of 10 slices across 200 *μ*m depth were taken (each slice is 22 *μ*m apart along the axial direction). Each *x*-*y* scan slice of the *z*-stack image was 1024 × 1024 pixels with pixel size of 0.8 *μ*m and pixel dwell time of 50 *μ*s/pixel. The lowest slice was taken right above the glass surface of culture dishes (slice 1 in the upper left panel of Figure 1) and went all the way up to 200 *μ*m above (slice 10 in the upper left panel of Figure 1). 

### 2.4. Image Analysis—Image Spatial Correlation

The spatial correlation function below (1) was then applied to each *x*-*y* plane of SHG images:

(1) $$\begin{array}{c} G_{s}\left(\xi ,\psi \right)=\frac{{\langle I\left(x,y\right)I\left(x+\xi ,y+\psi \right)\rangle }_{x,y}}{{\langle I\left(x,y\right)\rangle }_{x,y}^{2}}-1, \end{array}$$

where *I* is the intensity, *ξ* and *ψ* are the spatial increments in the *x* and *y* directions, respectively, and the angle bracket indicates the average over all the spatial locations in both *x* and *y* directions. Since smaller scale structures are not the subject of this study, the original 1024 × 1024 pixel images were binned 4 × 4, which reduce the images to 256 × 256 pixels. The image spatial correlation was computed for the binned 256 × 256 image. After binning, the correlation image corresponds to pixel size of 3.2 *μ*m. More description of this method can be found at [20, 22]. The binning and spatial correlation analysis was done by SimFCS software developed at the Laboratory for Fluorescence Dynamics (available at http://www.lfd.uci.edu/).

### 2.5. Image Analysis—Estimation of the Collagen Compaction Level

In this study, we have found no specific directionality of collagen fibers and organization, which results in symmetrical spatial correlation images along both *x* axis and *y* axis (see, e.g., Figure 1, the lower left panel of the correlation image). Hence, the cross-sections of *x*-axis and *y*-axis were used for fitting.

A script was written in MATLAB (MathWorks, Natick, MA) to perform a fit of the cross-section of the image spatial correlation function. Figure 1 lower right panel shows that we need at least 2 Gaussian functions for the fitting, one very narrow that corresponds to the size of the pixel (the green dashed curve in Figure 1 lower right panel) and a broad Gaussian which corresponds to the low spatial frequency fluctuation of the collagen matrix (the blue dashed curve in Figure 1 lower right panel). The amplitude and standard deviation of the broad Gaussian (*A*, *σ*) were extracted. The average peak amplitude of low spatial frequency components is 4.5 (the average of *A*). For the images with *A* smaller than 0.001, the value of *σ* cannot be reliably measured. In the following, we mainly discuss the *σ* value, which reports the mesoscale collagen spatial compaction level. Note that in this repot we used the Gaussian fit not as a model of the spatial frequency fluctuations but as an algorithmic approach to estimate the spatial extent of the SHG image structure.

### 2.6. Data Plotting and Statistics

Data plotting was done using MATLAB. For each data group, the highest and lowest *σ* values were removed before analysis. The error bars on all graphs were one standard deviation. Two-sample *t*-test was used to compare data. We report the data as significantly different if the *P* value is smaller than 0.01. 

## 3. Results

To evaluate the impact of ECM remodeling by MCF-7, NIH/3T3, and MDA-MB-231 cells, type I collagen matrices with cells embedded were imaged using two-photon microscopy after one, two, and four days of collagen polymerization. SHG *z*-stack images covered 200 *μ*m depth (10 *z* slices in total) and 819 *μ*m of both width and length were collected and analyzed as shown in Figure 1. Collagen matrix with no cells was prepared under the same polymerization and incubation conditions for baseline comparison. 

**Figure 2:**
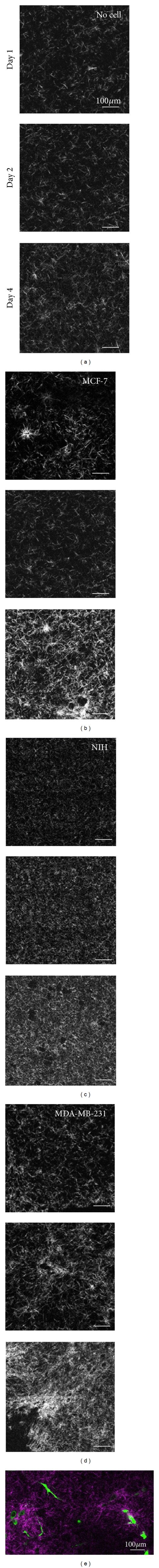
*Representative SHG images of collagen matrix with and without cells*. (a) Type I collagen matrix without cells after 1, 2, and 4 days of incubation. There is no recognizable collagen fiber distribution heterogeneity or collagen compaction. (b) Collagen matrix with MCF-7 cells after 1, 2, and 4 days of incubation. There is no distinguishable feature until day 4, in which collagen matrix with MCF-7 cells shows mild remodeling, although the majority of the collagen matrix was not compacted by cells. (c) Collagen matrix with NIH/3T3 cells after 1, 2, and 4 days of incubation. A higher degree of collagen compaction compared to the collagen matrix with MCF-7 cells can be seen since day 2. Higher collagen density was also observed, possibly due to collagen synthesis by the fibroblasts. (d) Collagen matrix with MDA-MB-231 cells after 1, 2, and 4 days of incubation. Collagen matrix shows significantly higher degree of remodeling compared to both MCF-7 and NIH/3T3 cell-embedded collagen matrices. (e) Actin-GFP labeled MDA-MB-231 cells cultured in type I collagen matrix for 4 days, showing that collagen matrix (magenta) has higher density close to cells (green). The higher density area around the cell is referred to as collagen compaction, and its size is quantitatively evaluated by image spatial correlation in this report.

Figures 2(a)–2(d) shows the collagen matrix SHG images of collagen alone, collagen with MCF-7, NIH/3T3, and MDA-MB-231 cell line, respectively. Contrary to the relatively homogenous collagen fiber distribution from collagen matrix without cell seeding shown in Figure 2(a), collagen organization heterogeneity can be clearly seen after one day of MDA-MB-231 cell seeding, and the collagen compaction level at some local regions increased over time (Figure 2(d)). On the other hand, the less-invasive breast cancer cell line MCF-7 and fibroblast cell line NIH/3T3 have more mild effects on collagen compaction level (Figures 2(b) and 2(c)).

**Figure 3:**
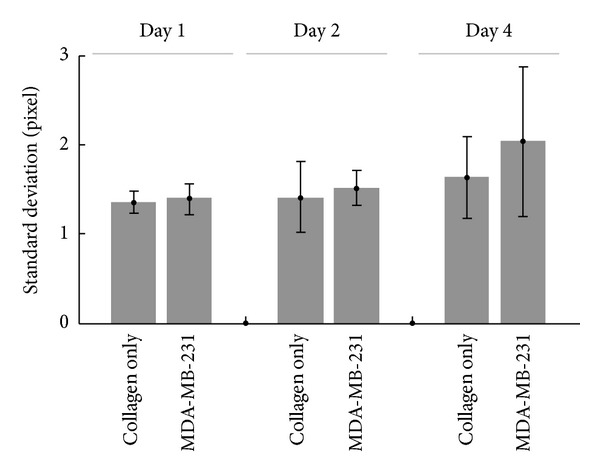
*The standard deviation of narrow peak is not significantly affected by collagen remodeling*. For the collagen matrix with or without MDA-MB-231 cells embedded, the standard deviation of the narrow peak from spatial correlation images (the green curve in Figure 1) did not show any significant difference, and its value was close to the pixel size.

The association of the high collagen matrix density region with the cell locations can be seen in Figure 2(e), where the collagen fibers (magenta) were densely packed around MDA-MB-231 cells (green). Our aim is to quantitatively assess the average size of these pericellular collagen compactions due to cell-ECM remodeling. As described in the method section, to capture this mesoscale collagen matrix feature, we calculated image spatial correlation of SHG images (Figure 1) to statistically estimate the average size of collagen compaction. The image spatial correlation gives rise to a sharp peak, which corresponds to the pixel size, and a broader distribution, which reflects the actual size of the structure. The lower right panel in Figure 1 represents an example of spatial correlation with this property. To identify the characteristic scale of these two distinct spatial frequency features, we used two Gaussian components fitting to the spatial correlation images. The standard deviation value of the sharp Gaussian of the SHG spatial correlation, as shown in Figure 3, was not affected by cell ECM remodeling. It coincides with the value of the pixel size and does not have significant change over time. In contrast, the standard deviation of low frequency component reflects the average size of the collagen compaction, and the amplitude is a function of signal-to-background ratio and of the proportion of the broad Gaussian. 

Here we define *σ* as the standard deviation of low spatial frequency component (i.e., the broader distribution), which in our experiment was in the order of tens of micrometers. Within 4 days, the average *σ* of collagen matrix with MDA-MB-231 cells increases significantly from 17 *μ*m in day 1, 21 *μ*m in day 2, to 39 *μ*m after 4 days of incubation (Figure 3). The average *σ* of collagen matrix without cells shows much smaller change, increasing by only 1 *μ*m after 4 days compared to ~20 *μ*m increase in the presence of MDA-MB-231 cells. Consistent with the hypothesis that less invasive tumor cells may cause less collagen compaction, we found that the collagen SHG images with MCF-7 cells embedded showed significantly smaller *σ*. The collagen remodeling ability of NIH/3T3 cell line lies between MCF-7 and MDA-MB-231 cell line. However, unlike MDA-MB-231 cell line, the change of *σ* over time was minimal for NIH/3T3 embedded collagen matrix.

We exclude the possibility of the value of *σ* due to the space occupied by cells based on the following. (1) The size indicated by *σ* is larger than cell body; (2) cell size does not change over time, but collagen compaction level increases dramatically over time; and (3) the cell size is comparable for the three cell lines examined.

To further assess the method's applicability to evaluate collagen remodeling, we performed Latrunculin B treatment and Marimastat treatment to MDA-MB-231 cells cultured in the type I collagen matrix. Latrunculin B affects the actin polymerization degree, which leads to the altered mechanical properties of the cell, including decreased contractile force [23]. Marimastat is a broad-spectrum matrix metalloproteinase inhibitor that blocks the activity of MMP-1, 2, 3, 7, 9, and 12 [24] and has been shown to affect collagen remodeling *in vitro*. With either Latrunculin B or Marimastat treatment, the collagen compaction level by MDA-MB-231 cells was significantly hampered (Figure 5). The treatment with Marimastat decreased the magnitude of collagen remodeling from the average *σ* = 37 *μ*m to *σ* = 29 *μ*m, while Latrunculin B showed even more severe effect that leaded to *σ* similar to MCF-7 cell line (*σ* = 21 *μ*m).

**Figure 4:**
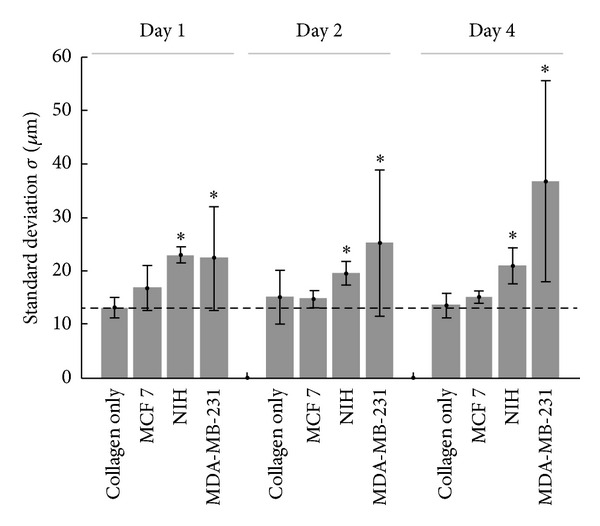
*Comparison of collagen remodeling degree with different cell lines*. The degree of collagen remodeling by MCF-7, NIH/3T3, and MDA-MB-231 cell lines over 4 days of incubation was evaluated by *σ* of the low spatial frequency component (the blue curve in Figure 1). Of all three cell lines, MDA-MB-231 showed the highest degree of collagen remodeling, and the collagen compaction degree increases over 4-day period. MCF-7 exhibits the lowest degree of collagen compaction and has the *σ* value similar to the collagen matrix without cells. Dashed line shows the average *σ* value of collagen matrix without cells at day 1. * indicates that the standard deviation is significantly different from the baseline control (collagen matrix without cells for each equivalent age of the sample, *P*  value < 0.01 by *t*-test).

**Figure 5:**
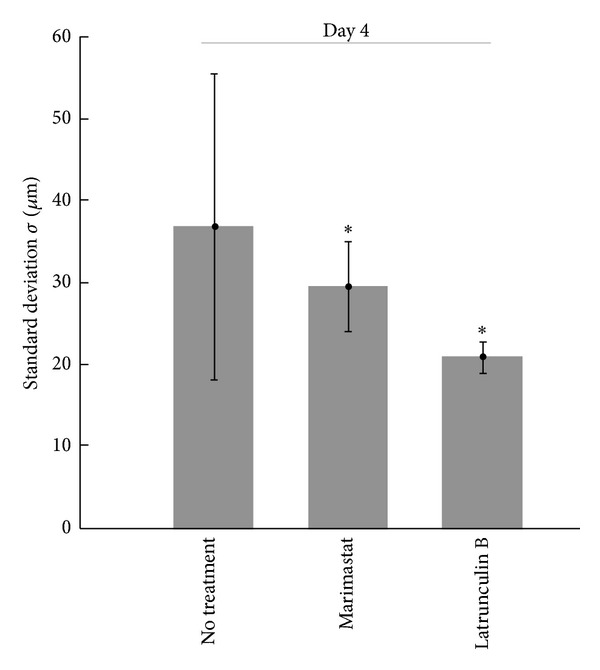
*MMP inhibitor and actin polymerization inhibitor affect MDA-MB-231 collagen remodeling ability*. MDA-MB-231 cells were cultured in collagen matrix and treated with Marimastat (MMP inhibitor) or Latrunculin B (actin polymerization inhibitor) for 4 consecutive days. Both treatments significantly compromised the collagen compaction ability of MDA-MB-231 cells. While the effect of Marimastat is milder, Latrunculin B treatment resulted in the compaction degree similar to collagen matrix with MCF-7 cells. * indicates the standard deviation is significantly different from the collagen matrix without cells (*P*  value < 0.01 by *t*-test).

For a structure with given geometry in an image, the structure size is linearly correlated to the standard deviation of spatial correlation as obtained from the Gaussian fit. The resulting spatial correlation will have a standard deviation that is half of the original structure size due to the properties of the correlation function. The amplitude, on the other hand, is proportional to the area of the structure and signal-to-background ratio. The result from collagen SHG images used in this study, thus, represents a weighted average of complex patterns, with two major components: the pixel-size correlation due to the small collagen fiber width and its random orientation (Figure 3) and the collagen compaction area created by cell-matrix interaction, which is highly variable (Figures 4 and 5). Here, we demonstrated that by using spatial image correlation it is possible to quantify and differentiate the impact of cells on collagen matrix under different conditions. The estimated collagen compaction structure size from MDA-MB-231 cell after 4 days in collagen is around 70 *μ*m. Compared to an average cell body diameter of 20–30 *μ*m, the collagen structure emerging after cell seeding may be much larger than the single cell. 

## 4. Discussion

The interplay between the biophysical properties of the cell and ECM establishes a dynamic mechanical reciprocity between the cell and the ECM in which the cell's ability to exert contractile stresses against the ECM balances the elastic resistance of the ECM to deformation. In this sense, ECM is effectively a physical extension of the cell and cytoskeleton [25]. In this study, we described a method based on spatial correlation to quantify the degree of matrix remodeling at the tens of micron scale. We chose cell lines (MCF-7, NIH/3T3, and MDA-MB-231) that presumably could show a difference in regard to collagen compaction at the mesoscale. We were able to estimate the average collagen compaction area by MCF-7 cells, NIH/3T3 cells, and MDA-MB-231 cells. With the same initial type I collagen concentration, the more invasive cancer cell line, MDA-MB-231, was able to generate significantly larger collagen compaction areas, while the less invasive cell line (MCF-7) has a modest effect on the collagen matrix. The NIH/3T3 cell line exhibits the collagen remodeling ability between MCF-7 and MDA-MB-231 cell lines. This result is consistent with previous studies showing that tumor cell invasiveness is associated with increased contractile force generation [21], which affects the collagen remodeling ability. This notion was further supported by the significant decrease of *σ* when MDA-MB-231 cells were treated with Latrunculin B, which inhibits actin polymerization and decreases cellular contractility [23]. Blocking MMPs by Marimastat, although with smaller impact compared to the effect of actin cytoskeleton disruption, also showed significant alteration on the degree of collagen compaction. In fact, MMPs have been associated to tumor stroma and fibrosis *in vivo* [26, 27]. A recent research conducted by tracking bead displacement to assess ECM remodeling also showed the treatment of MMP inhibitor reduced the magnitude of ECM deformation [28], supporting our evaluation by spatial correlation method. 

High breast tissue density due to increased type I collagen is one of the single largest risk factors for developing breast cancer [29]. This higher stiffness correlates with increased mammographic density and has been exploited to detect cancer [4, 30–32]. Matrix stiffness from type I collagen cross-linking has also been implicated as a contributor to the enhanced invasive behavior in tumor [33], enhanced cell growth and survival, and promoted migration. However, what causes the increase in tumor stromal stiffness and how stromal stiffness contributes to tumor progression is still not clear. In addition, the causality of matrix stiffness and tumor invasiveness has yet to be determined. Our data indicate that tumor cells may be involved in ECM remodeling and contribute to the different degree of collagen compaction based on their invasiveness. This hypothesis is also supported by *in vivo* tumor collagen images which showed collagen stretching near invasive cancer cells [4], which is similar to what we observed *in vitro*. 

In this report, we showed that the image spatial correlation method provides a measure of ECM spatial organization, in this example the collagen compaction by cells, at the scale comparable or larger than the cellular size. The advantages of the analysis method proposed in this report include (1) the simple statistical procedure that does not require image segmentation or other predefined parameters, which could be more subjective; (2) it does not depend on the intensity range of each image acquired, so the comparison between different images can be achieved without difficulty; and (3) the use of SHG to image collagen fibers that does not require extra labeling and could be directly applied to *in vivo* studies. Due to its simplicity of implementation, this method has the potential to not only facilitate *in vitro* ECM remodeling study or ECM assessment for tissue engineering purposes but can be also applied to *in vivo* research for determining regional collagen organization differences in tissues and tumor sites. By choosing the proper pixel size, it is possible to use this method to detect structures of different scales and to quantify each component's significance. Provided there is enough axial resolution, image spatial correlation can be used in three-dimensions, making it possible to tell whether collagen exhibits different spatial organization along lateral and axial directions. Furthermore, although not shown in this report, the directional matrix alignment could be estimated by 2D Gaussian fitting to the spatial correlation image, which may increase the application of this method.
